# Preoperative CSF Melatonin Concentrations and the Occurrence of Delirium in Older Hip Fracture Patients: A Preliminary Study

**DOI:** 10.1371/journal.pone.0167621

**Published:** 2016-12-09

**Authors:** Rikie M. Scholtens, Sophia E. J. A. de Rooij, Annelies E. Vellekoop, Bart C. Vrouenraets, Barbara C. van Munster

**Affiliations:** 1 Academic Medical Center, University of Amsterdam, Department of Internal Medicine, Geriatrics section, Amsterdam, The Netherlands; 2 University Medical Center Groningen, University Centre for Geriatric Medicine, Groningen, The Netherlands; 3 Erasmus Medical Center, Department of Urology, Rotterdam, The Netherlands; 4 Sint Lucas Andreas Hospital, Department of Surgery, Amsterdam, The Netherlands; 5 Gelre Hospitals, Department of Geriatrics, Apeldoorn, The Netherlands; University of Glasgow, UNITED KINGDOM

## Abstract

**Background:**

Delirium is characterized by disturbances in circadian rhythm. Melatonin regulates our circadian rhythm. Our aim was to compare preoperative cerebrospinal fluid (CSF) melatonin levels in patients with and without postoperative delirium.

**Methods:**

Prospective cohort study with hip fracture patients ≥ 65 years who were acutely admitted to the hospital for surgical treatment and received spinal anaesthesia. CSF was collected after cannulation, before administering anaesthetics. Melatonin was measured by radioimmunoassay (RIA). Data on delirium was obtained from medical and nursing records. Nurses screened every shift for delirium using the Delirium Observation Screening Scale (DOSS). If the DOSS was ≥3, a psychiatrist was consulted to diagnose possible delirium using the DSM-IV criteria. At admission, demographic data, medical history, and information on functional and cognitive status was obtained.

**Results:**

Seventy-six patients met the inclusion criteria. Sixty patients were included in the analysis. Main reasons for exclusion were technical difficulties, insufficient CSF or exogenous melatonin use. Thirteen patients (21.7%) experienced delirium during hospitalisation. Baseline characteristics did not differ between patients with and without postoperative delirium. In patients with and without postoperative delirium melatonin levels were 12.88 pg/ml (SD 6.3) and 11.72 pg/ml (SD 4.5) respectively, *p*-value 0.47. No differences between patients with and without delirium were found in mean melatonin levels in analyses stratified for cognitive impairment or age.

**Conclusion:**

Preoperative CSF melatonin levels did not differ between patients with and without postoperative delirium. This suggests that, if disturbances in melatonin secretion occur, these might occur after surgery due to postoperative inflammation.

## Introduction

Delirium is a neuropsychiatric syndrome that is highly prevalent in older persons admitted to the hospital.[1] Patients with dementia, postoperative patients and persons with critical illnesses are at increased risk of developing delirium.[2] Not only is delirium associated with prolonged hospital stay and higher health care costs, it is also associated with long term functional and cognitive decline.[3, 4] An important feature of delirium is that it fluctuates over the course of the day.[5] Clinicians often observe sleep and wake cycle disturbances in delirious patients. Currently, the exact underlying pathophysiology of delirium is unknown. Several mechanisms, such as disturbances in neurotransmitters, (neuro)inflammation, oxidative stress, and diurnal dysregulation have been proposed.[6, 7] Melatonin is known to be a key factor in the regulation of circadian rhythm and thus could play a role in diurnal rhythmicity disruptions.[8] Melatonin has a distinct secretion pattern with low levels during the day and a maximal concentration between 01:00 and 04:00 am.[8] This pineal hormone is released directly into cerebrospinal fluid via the pineal recess in the third ventricle and in blood via the vein of Galen.[9–11]

Recent studies found that plasma melatonin levels differed in patients with dementia, after surgery and with ageing in general.[12–14] In older adults, melatonin levels were found to be lower than in younger persons.[13, 15] Changes in the secretion pattern and lower melatonin levels were found in patients with dementia compared to patients without dementia.[14] Postoperative nocturnal melatonin levels were lower during the first three nights after surgery compared to nocturnal melatonin levels before surgery.[12] As the abovementioned factors are known to increase the risk of developing delirium, one could hypothesize that melatonin levels might also alter in patients experiencing delirium. Previous research on plasma melatonin levels during delirium showed lower maximal levels during delirium than after delirium resolution or in patients without delirium. [16, 17] This could indicate that melatonin supplementation might be beneficial for patients at risk for delirium. However, previous studies on melatonin supplementation for the prevention of delirium have been contradictory and showed no clear beneficial effect on delirium incidence.[18]

The majority of studies have measured melatonin in blood. Because of the proximity to the pineal gland and the brain itself, cerebrospinal fluid (CSF) measurements are an interesting target for research and could perhaps better reflect cerebral processes. As melatonin is released directly into CSF, this could provide more accurate information on melatonin production and secretion. Presently, little is known about human CSF melatonin levels. Research in CSF and brain tissue of diseased patients showed that patients with Alzheimer’s disease had lower levels of melatonin in CSF and in brain tissue compared to healthy controls.[19–21] Also, melatonin levels were lower in patients aged 80 years or older when compared to patients younger than 80 years. In patients with traumatic brain injury, morning CSF melatonin levels were 7.3 pg/ml versus 1.5 pg/ml in controls.[22] Bumb et al. measured CSF melatonin levels in young and healthy volunteers before and after sleep deprivation and found higher morning levels after sleep deprivation (7.7 pg/ml vs 3.2 pg/ml).[23] Due to ethical and practical reasons, no studies have been conducted that studied the daily CSF melatonin profile in (older) persons. In mammals a distinct secretion pattern was observed, with higher CSF melatonin levels at night compared to plasma levels and low CSF daytime levels.[24]

Elderly patients with a hip fracture are prone to develop delirium after surgery and commonly receive spinal anesthesia whereby CSF can be obtained without an additional invasive procedure. If alterations in CSF melatonin levels were associated with higher delirium incidence, this would provide more insight into delirium pathophysiology. No CSF melatonin concentrations are known in patients with delirium. Therefore, the aim of this study was to compare preoperative CSF melatonin levels in elderly patients with and without subsequent (postoperative) delirium.

## Methods

### Study design and patients

We conducted a prospective cohort study. From March 2012 to April 2014, individuals aged 65 years and older who were acutely admitted for surgical repair of a hip fracture to the Sint Lucas Andreas Hospital in Amsterdam were recruited. Written informed consent was obtained from the patient or legal representative in case of incompetence. Patients who did not receive spinal anesthesia, were unable to speak or understand Dutch or English, or left the surgical ward within 48 hours after admission, were excluded. The choice for general or spinal anesthesia was at the doctors’ and patients’ discretion and based upon the hospital’s protocol. The Medical Ethics Committee of the Academic Medical Center Amsterdam approved the study protocol. The study was conducted according to the Good Clinical Practice guidelines and in compliance with the Declaration of Helsinki.

### Baseline and clinical characteristics

Demographic data, medication use at home and medical history were collected at admission. Functional decline was measured by the 6-item KATZ Index of Activities of Daily Living in which the current status was compared to the situation two weeks prior to admission with a maximal score of 6.[25]The primary caregiver filled in the Informant Questionnaire on Cognitive Decline Short Form (IQCODE-sf) and was asked to compare the current situation with 10 years earlier.[26] Cognitive impairment was defined as an IQCODE-sf > 3.4 or diagnosed dementia in the medical history. The Charlson Comorbidity Index was calculated to describe comorbidity.[27]

### Assessment of delirium

Nurses screened all patients 3 times a day (once every shift) for delirium using the Delirium Observation Screening Scale (DOSS) seven days a week. In the Netherlands, delirium screening with the DOSS is recommended in the Dutch delirium guideline and is part of daily clinical practice in most hospitals.[28, 29] A cut-off score of ≥3 on the DOSS was used to indicate delirium. A recent review on instruments for delirium detection showed that the Delirium Observation Screening Scale is a validated tool used by nurses with good predictive validity against the DSM-IV diagnosis of delirium made by a geriatrician, with high sensitivity and specificity for delirium, and easy to apply in daily clinical practice at the bedside. [30] The scale has been translated in English and Dutch. [31] Information about delirium symptoms from medical and nursing records considering the previous 24 hours was taken into account as well. If delirium was suspected, a psychiatrist was consulted to perform a full mental examination and to evaluate the patient for delirium using the criteria of the fourth edition of the Diagnostic and Statistical Manual of Mental Disorders (DSM-IV-TR) [5]

### Cerebrospinal fluid collection and melatonin measurement

Surgery was performed daily and was not limited to office hours. CSF was collected after cannulation for the introduction of spinal anesthesia, prior to administering anesthetics. CSF samples were immediately brought to the laboratory. After centrifugation, the samples were alliquoted and stored at -80°C until melatonin measurement. Melatonin was measured in duplicate by a direct radioimmunoassay (Melatonin Research RIA, Labor Diagnostika Nord GmbH & Co, Germany). If the two measurements differed < 12.5% from each other (the coefficient of variation of the RIA), we took the mean of the measurements. If the measurements differed > 12.5%, we did not use these and performed a new measurement in duplicate. The lower detection limit of the assay was 3 pg/ml.

### Statistical analysis

All analyses were performed using SPSS version 22.[32] Descriptive statistics were used to report demographic and clinical data. Baseline differences between patients with and without postoperative delirium were assessed with an unpaired T-test, χ^2^ test, Fisher’s exact test or Mann-Whitney U test, as appropriate. Differences in melatonin level between groups were assessed with an unpaired T- test or with an one-way ANOVA with Bonferroni post-hoc tests. To assess the correlation between age and CSF melatonin levels, a Spearman’s rank correlation test was used to avoid normal distribution assumptions of the data. We determined the season when the sample was collected to control for their influence as melatonin levels might be lower in winter compared to summer. We defined season by the month of surgery. Summer was defined as June, July or August, autumn as September, October or November, winter as December, January or February and spring as March, April or May.

## Results

### Descriptive statistics

**Fig 1:**
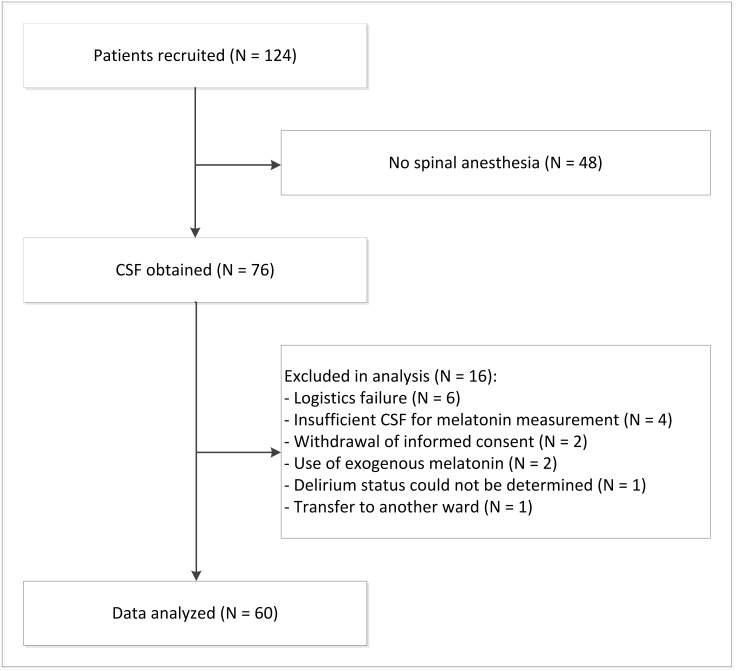
Flow chart of the selection of patients.

In total, 250 consecutive patients underwent hip surgery from March 2012 to April 2014 of which the vast majority were over the age of 65 years old. One hundred and twenty-four patients were enrolled in our study. Of these enrolled patients, 76 persons received spinal anesthesia and provided CSF. Sixty patients were included in the analysis. Main reasons for exclusion were that samples could not be identified, exogenous melatonin use or insufficient CSF for melatonin measurement. (Fig 1) Of these 60 patients, 13 experienced delirium (21.7%). Twelve patients were diagnosed with delirium after surgery: six patients on the same day as surgery was performed, four patients one day after surgery, one patient two days after surgery and one patient six days after surgery. One person was diagnosed with delirium before surgery and left out of further analyses. Most patients had surgery on the day of admission (N = 19) or the day after admission (N = 34). Baseline characteristics did not differ between patients who experienced postoperative delirium and those who did not experience delirium. (Table 1)

**Table 1:** Characteristics of hip fracture patients with and without postoperative delirium. Characteristics are shown as number of patients (%) unless stated otherwise.

	Postoperative delirium	No postoperative delirium	*p*-value
	N = 12	N = 47	
**Mean age in years (SD)**	86.4 (8.0)	83.4 (7.7)	0.24
**Sex male**	2 (16.7)	16 (34.0)	0.31
**Living at home**	8 (66.7)	35 (74.5)	0.69
**Social status**			0.30
Living alone	9 (75.0)	22 (46.8)	
Living together with partner/child	2 (16.7)	13 (27.7)	
missing	1 (8.3)	12 (25.5)	
**Median preadmission KATZ-ADL-6 score (q1-q3)**	4 (0–5)	1.5 (0–4)	0.30
missing	0 (0.0)	7 (14.9)	
**Median Charlson Comorbidity Index (q1—q3)**	2 (1–3)	2 (1–4)	0.97
**Premorbid cognitive impairment**	3 (25.0)	11 (23.4)	1.00
**Type of fracture**			0.62
intertrochanteric	6 (50.0)	27 (57.4)	
femoral neck	6 (50.0)	16 (34.0)	
other	0 (0.0)	4 (8.5)	
**Type of surgery**			0.44
internal fixation	8 (66.7)	38 (80.9)	
hip replacement	4 (33.3)	9 (19.1)	
**Median time to surgery in days (q1-q3)**	1 (0.3–1)	1 (0–1)	0.61
**Time of obtaining CSF**			0.82
24:00–06:00	0 (0.0)	0 (0.0)	
06:00–12:00	2 (16.7)	10 (21.3)	
12:00–18:00	7 (58.3)	27 (57.4)	
18:00–24:00	3 (25.0)	8 (17.0)	
missing	0 (0.0)	2 (4.3)	
**Season of obtaining CSF**			0.56
spring	4 (33.3)	13 (27.7)	
summer	2 (16.7)	14 (29.8)	
autumn	5 (41.7)	12 (25.5)	
winter	1 (8.3)	8 (17.0)	
**Overall mean CSF melatonin level in pg/ml (SD)**	12.88 (6.3)	11.72 (4.5)	0.47
**Mean CSF melatonin level stratified for time of the day of sampling in pg/ml (SD)**			
06:00–12:00	9.30 (1.0)	9.05 (4.1)	0.94
12:00–18:00	11.71 (3.5)	12.97 (4.0)	0.45
18:00–24:00	18.00 (11.1)	10.13 (5.1)	0.13

q1-q3 = first quartile—third quartile, CSF = Cerebrospinal Fluid, SD = Standard Deviation, ADL = Activities of Daily Living

### CSF melatonin concentrations

**Fig 2:**
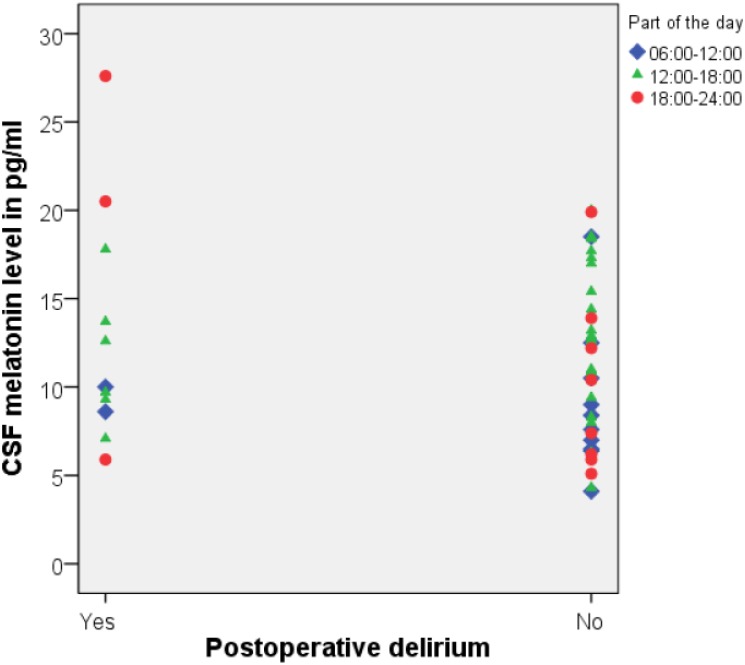
Preoperative CSF melatonin levels in pg/ml in patients with and without postoperative delirium.

In all samples, the melatonin concentrations were within the detection limit of the assay. Mean preoperative CSF melatonin level was 11.87 pg/ml (SD 4.9 pg/ml). In patients with postoperative delirium, melatonin levels were 12.88 pg/ml (SD 6.3 pg/ml) and in patients without postoperative delirium, melatonin levels were 11.72 pg/ml (SD 4.5 pg/ml), *p*-value 0.47. (Fig 2) One patient that was delirious at the time of sampling, had a melatonin level of 6.5 pg/ml. We could not demonstrate an effect for the time of the day or season when CSF samples were obtained. Age was not correlated with CSF melatonin levels. (Spearman’s ρ: 0.20, *p* = 0.13). No differences in mean melatonin levels were found between patients who experienced postoperative delirium and those who did not in analyses stratified for cognitive impairment.

## Discussion

To the best of our knowledge, this is the first study that measured melatonin in CSF in older hip fracture patients and evaluated the association of CSF melatonin levels with delirium. We could not demonstrate a difference in preoperative CSF melatonin levels in older hip fracture patients that experienced postoperative delirium compared to older hip fracture patients who did not. This suggests that the melatonin level is not different for patients at risk for delirium.

Alterations in melatonin secretion or secretion pattern may cause older persons to develop delirium or possibly even induce delirium.[7] Cronin and colleagues showed lower plasma melatonin levels at night in the first three nights after surgery compared to plasma melatonin levels before surgery.[12] Moreover, alterations in melatonin secretion pattern were described in postoperative patients with delirium who previously had a normal secretion pattern. [17] No studies on perioperative CSF melatonin levels have been performed to determine if possible changes in CSF melatonin secretion might occur after surgery due to postoperative inflammation.

Mean CSF melatonin levels in our study were 11.87 pg/ml. Previous studies reported CSF concentrations from 1.47 pg/ml up to 542 pg/ml in physiological conditions.[22, 33–37] The wide range of CSF melatonin levels are mostly due to the variation in measurement techniques used and brain compartment where CSF samples are obtained. When CSF melatonin levels of the present study were compared to studies that collected CSF at the lumbar cistern, the levels were similar. [23, 35, 36]

### Limitations

Due to the preliminary nature of this study and small number of patients with delirium, no multivariate analysis was performed. However, analyses stratified for the most important confounders, age and cognitive impairment did not show differences in CSF melatonin levels. In our population, baseline characteristics that were associated with a higher risk of developing delirium, like higher age and cognitive impairment, did not differ between patients who experienced delirium and patients who did not. This could indicate that the number of patients experiencing delirium in our study was too small.

CSF samples were obtained under bright light conditions and under stressful circumstances for the patient. Although the time to surgery was short, hospital stay and experiencing pain could already have induced sleep disturbances to some extent in any of our patients. These factors could have confounded our findings and endorse the need for CSF measurements in non-surgical populations and for determination of physiological CSF melatonin levels in elderly. More research is required to further explore our preliminary findings.

In this study, a CSF sample was obtained before surgery and delirium usually was diagnosed within 48 hours after surgery. Therefore, differences in CSF melatonin concentrations may have been missed due to the time between sampling and the occurrence of delirium. No information about the secretion pattern of melatonin could be obtained because we were only able to obtain one CSF sample due to practical and ethical reasons. As circadian rhythm disturbances frequently occur in delirium, alterations in melatonin concentrations could happen later on in the disease process and preoperative sampling may be premature to see meaningful change. No conclusions about CSF melatonin levels during delirium could be drawn as only one person was delirious at the time of CSF sampling. Timing of sampling appears very important and could have been a cause for not finding an association between CSF melatonin levels and postoperative delirium in this study.

Overall, the incidence of delirium was 21.7% in this study which is line with previous studies describing delirium incidence in postoperative older people.[1, 38] The time to assess patients before surgery was limited, which might have led to underestimation of the delirium incidence preoperatively as delirium is a 24 hours diagnosis and symptoms fluctuate over the day. Besides this, delirium is a difficult diagnosis and could not have been recognized in all cases by medical and nursing staff, resulting in not consulting the psychiatrist. The hypoactive subtype of delirium or delirium superimposed on dementia are sometimes difficult to diagnose by staff that is not trained in geriatrics or psychiatrics and the sensitivity of the DOSS might be insufficient for this matter.[39] The DOSS has not been validated for delirium screening in different motor subtypes, nor for delirium superimposed on dementia. Underreporting of delirium could be an explanation for not finding a difference in melatonin levels between delirious and non-delirious patients.

### Future research

Future research should focus on melatonin levels in the perioperative period, and repeated sampling before, during and after delirium. Also the relationship between CSF melatonin levels and blood melatonin levels must be further explored to see if blood levels correlate with CSF melatonin concentrations. To further explore our preliminary findings, future research should include a larger sample size to exclude a type 2 error, diagnose delirium according to the DSM criteria, and include measurements in the non-surgical population.

## Supporting Information

S1 File SPSS dataset for Preoperative CSF melatonin levels and the occurrence of delirium in older hip fracture patients: a preliminary study. (SAV)
